# Altered shear stress on endothelial cells leads to remodeling of extracellular matrix and induction of angiogenesis

**DOI:** 10.1371/journal.pone.0241040

**Published:** 2020-11-19

**Authors:** T. A. Russo, A. M. M. Banuth, H. B. Nader, J. L. Dreyfuss

**Affiliations:** Molecular Biology Division, Department of Biochemistry, Carl Peter von Dietrich Laboratory, Escola Paulista de Medicina, Universidade Federal de São Paulo, São Paulo, Brazil; University of Sharjah College of Health Sciences, UNITED ARAB EMIRATES

## Abstract

Endothelial cells (ECs) are subjected to physical forces such as shear stress (SS) induced by blood flow that leads to significant changes in morphology, physiology and gene expression. The abnormal mechanical forces applied in the cardiovascular system can influence the development of conditions and diseases such as thrombosis, hypertension and atherosclerosis. This study investigated the expression of glycosaminoglycans (GAGs), proteoglycans and extracellular matrix molecules in ECs exposed to normal and altered SS. ECs were exposed to SS of 12 dyn/cm^2^ (artery physiological condition) and 4 dyn/cm^2^ (artery pathological condition). Subsequently, ECs were subjected to immunofluorescence, qPCR, GAG biosynthesis analyses and cell-based assays. SS induced changes in ECs morphology. There were other pathological consequences of altered SS, including inhibited adhesion, stimulation of migration and capillary-like tube formation, as well as increases of GAG synthesis. We observed higher expression of syndecan-4, perlecan, decorin, fibronectin and collagen III α1 and growth factors, including VEGF-A and TGFβ-1. ECs exposed to SS displayed extracellular matrix remodeling as well as expression of cell-matrix and cell-cell interaction molecules. This study contributes to the understanding of how vascular biology is affected by mechanical forces and how these molecules can be affected in cardiovascular diseases.

## Introduction

Vascular endothelial cells (ECs) are exposed to mechanical forces such as stretching, tension, compression and shear stress that modulate their functional properties; this phenomenon is known as mechanotransduction, and it may be physiologic or pathologic [1, 2]. Mechanotransduction is the conversion of mechanical forces into molecular and cellular responses [3]. This usually results in intracellular changes to compensate for the stress. Blood flow regulates the internal diameter of the arteries in two ways: by constricting and relaxing vascular smooth muscle cells and by reorganizing the vessel wall (ECs and extracellular matrix) [1, 4]. In both cases, the endothelium is the key factor, acting as a mechanical sensor. Cell components such as glycocalyx and extracellular matrix (ECM) interact with the cytoskeleton and are activated by mechanical deformation [5].

Shear stress is critically important in regulating normal atheroprotective physiology of the vessel, as well as inducing pathological changes and dysfunctions that promote atherogenesis if the flow is altered. Abnormal mechanical forces induce cellular responses that may occur in cardiovascular diseases such as hypertension, thrombosis, atherosclerosis and vessel wall inflammation. Moderate or high-intensity laminar shear stress may be atheroprotective [6]. Non-physiological flow alters gene expression, cytoskeletal arrangement, healing and leukocyte adhesion, as well as the oxidative and inflammatory responses of the artery wall. In disturbed or oscillatory flows near arterial bifurcations, branches and curvatures are associated with the formation of atherosclerotic plaques. Nevertheless, the exact mechanism of the association between systemic arterial hypertension and atherosclerosis is not fully understood [7]. Frequently, atherosclerosis is related to traditional risk factors such as systemic arterial hypertension, diabetes mellitus, increased levels of cholesterol, smoking and obesity. Notwithstanding the fact that the pathophysiology of atherosclerosis is a complex and multifactorial process, the role of shear stress on ECs has emerged as an essential feature of atherogenesis. Atherosclerotic plaques tend to form predominantly in regions of low shear stress, such as at flows of 4 dyn/cm^2^ that are investigated in this study, while regions of moderate- or high-intensity shear stress are generally protected [8].

When performing in vitro experiments to study EC mechanotransduction, it is important to mimic forces to which the endothelium is exposed in vivo. While conventional cell culture neglects mechanical force effects, shear stress can be mimicked in vitro using parallel chamber flow systems. In this study, shear stress was evaluated under artery physiological conditions, represented by forces of 12 dyn/cm^2^ (438 mL/min) and artery pathological conditions using a force of 4 dyn/cm^2^ (146 mL/min), both produced using laminar flow [9–12].

The understanding of mechanotransduction process and the role of this mechanical force on the expression of glycosaminoglycans (GAGs), proteoglycans (PGs) and other ECM molecules may indicate how changes in blood flow influences the behavior of ECs, consequently providing a better understanding of endothelial diseases.

## Materials and methods

All methods were carried out in accordance with relevant guidelines and regulations. All experimental protocols were approved by Comitê de Ética em Pesquisa—Universidade Federal de São Paulo (Research Ethics Committee)—CEP 373/12.

### Endothelial cell culture

Rabbit aortic endothelial cells (EC-clone CIPs), an immortalized cell culture were kindly provided by Dr. Vincenzo Buonassisi [13, 14]. This cell line has been a model in our laboratory for over 30 years [14–20] and this cell line has many endothelial characteristics including blood compatibility, expression of von Willebrand factor, tissue factor pathway inhibitor, and estrogen receptor, production of anticoagulant heparan sulfate, and fibronectin as well as development of capillary-like structures in vitro [14, 21–29]. The endothelial cells (ECs), 8^th^ passage, were maintained in F12 medium (Cultilab, Campinas, SP, Brazil) supplemented with 10% fetal bovine serum (Gibco®), streptomycin and penicillin (both 100 IU/ml) (Sigma-Aldrich, St. Louis, MO) and 20 mM sodium bicarbonate at 37°C in a humidified atmosphere (2.5% CO_2_) [14]. ECs were maintained in static conditions or exposed to different shear stress as describe below.

### Exposure of ECs to shear stress

ECs (3 x 10^4^ cells/mL) were plated on glass slides measuring 75 x 25 x 1 mm designed for the Streamer™, a device that uses parallel chambers with laminar flow to generate shear stress (Flexcell International Corp., Burlington, NC), coated with 1% sterile gelatin solution for 16 hours for better adherence (37°C, 2.5% CO_2_) until confluence. The system is designed to allow application of fluid flow in a sterile environment. The fluid perfused was F12 cell culture medium containing 10% fetal bovine serum. The apparatus was operated in a 2.5% CO_2_ incubator at 37°C. Shear stress (SS) was evaluated under artery physiological conditions using 12 dyn/cm^2^ and artery pathological conditions using 4 dyn/cm^2^, for 4 hours [10, 11]. ECs not exposed to SS were used as the static control. After EC exposure to shear stress or ECs in static culture were subjected to assays of gene expression of ECM components and growth factors, cell behavior assays and metabolic radiolabeling of GAGs were performed.

### Cell adhesion assay

The adhesion assay was performed in 96-well plates coated with 100 μl fibronectin (purified in our laboratory [30] from fresh human plasma, obtained from Hospital São Paulo–UNIFESP diluted in sterile phosphate-buffered saline (PBS) at various concentrations (0, 2.5, 5, 10, 15 and 20 μg/mL) for 18 hours at 4°C. Subsequently, the solutions were aspirated and the wells were rinsed with sterile PBS. Nonspecific binding sites were blocked with 150 μL bovine serum albumin (BSA) 5% in sterile PBS for 1 hour at 37°C, 2.5% CO_2_. Negative controls were performed in cell adhesion-sensitized wells only with BSA 5%. The BSA was aspirated and 5 x 10^4^ cells/well were plated in 100 μl of F-12 medium with 2% FBS and incubated for 2 h, 30 min at 37°C in 2.5% CO_2_. Unattached cells were removed by washing with PBS. Attached cells were fixed in methanol for 10 min, stained with 0.8% crystal violet (Sigma) dissolved in 20% ethanol for 15 min and washed 15 times in PBS. The dye was eluted with 50% ethanol in 0.1 M sodium citrate. The results were analyzed by reading the absorbance at 540 nm in an ELISA reader (Biochrom, Model 400 Microplate Reader EZ Read; Cambridge, England). The results were expressed as absorbance at 540 nm.

### Cell migration assay—Transwell®

5 x10^4^ ECs were plated in Transwell® inserts (6.5-mm diameter and 8-μm pore size) with 500 μl fresh culture medium F12 without FBS. To 24-well plates we added 800 μL of fresh culture medium F12 supplemented with FBS 10% (chemotactic agent). Then Transwell® inserts were placed over the wells. The cells were incubated at 37°C under 2.5% CO_2_ atmosphere for 12 hours. The wells were washed with PBS and non-migrating cells on the upper chamber removed using a cotton swab. Migrated cells were fixed with 4% paraformaldehyde and nuclei stained with DAPI. Image acquisitions were performed under an inverted light Axiovert 40 CFL microscope (Zeiss, Germany) and coupled camera (Nikon FDX) at 50X magnification. The results were calculated as total count number of nuclei stained with DAPI. For analyses and quantification of the images, we used ImageJ® Software (NIH, Bethesda, MD, USA).

### Capillary-like tube formation on reconstituted basement membrane assay

4 x 10^4^ ECs were plated in 70 μL of reconstituted basement membrane Geltrex^TM^ (Gibco®—Life Technologies, NY, USA) previously polymerized in 96-well plates, containing 100 μl of F-12 medium with 10% FBS. ECs were incubated for 8 hours at 37°C, 2.5% CO_2_. ECs were observed and photographed on an inverted light microscope (Primo Vert Monitor, Zeiss, Germany) at 100X and 400X magnification. The total number of tubular structures formed by ECs was measured using ImageJ® Software with the plug-in Angiogenesis Analyzer (NIH, Bethesda, MD, USA) for quantification analysis of the images. The results were expressed as the number of tubes formed as well as tube length (mm)/cm^2^.

### Immunofluorescence

ECs were immediately fixed with 4% formaldehyde for 20 min at room temperature. After being rinsed with PBS, cells were incubated with primary antibody rabbit anti-syndecan-4 (363100—Invitrogen^TM^, Life Technologies, USA), rabbit anti-connexin 43 (M-150—sc-9059—Santa Cruz Biotechnology, Texas, USA), or rabbit anti-perlecan (M-300—sc-25848—Santa Cruz Biotechnology, Texas, USA) in PBS containing 1% BSA overnight at 4°C. Subsequently, the cells were incubated with secondary antibody conjugated with fluorescent markers: Alexa Fluor 594 goat anti-rabbit (A11012), Alexa Fluor 488 chicken anti-mouse (A21200) or Alexa Fluor 488 goat anti-rabbit (A11008—Invitrogen^TM^, Life Technologies, USA), for 30 minutes in the dark. The nuclei were stained with DAPI (4'-6-diamidino-2-phenylindole, dihydrochloride) 1:10.000 (Molecular Probes) in PBS with 0.1% saponin for 30 minutes in the dark. The slides were mounted with a coverslip containing Fluoromount-G (EMS—Electron Microscopy Science, Washington, PA, USA). The slides were analyzed and the acquisition of the images were taken in a confocal microscope Leica TCS SP8 (Leica Microsystems, Wetzlar, Germany). Negative controls omitting the primary antibody are presented in S1 Fig.

### Synthesis of [^35^S]-sulfate glycosaminoglycans

The medium of ECs containing FBS was aspirated, the cells were washed with F12 medium without serum, and metabolic radiolabeling was carried out. For this, 1 ml of F12 medium without serum containing 150 μCi/mL [^35^S]-sulfate (POLATOM, Otwock, Poland) was added to each well and incubated for 18 hours at 37°C, 2.5% CO_2_. The culture-conditioned medium was collected and the cells were washed twice with PBS. The cell fraction was obtained by scraping the cells and extracellular matrix from the glass slide with 500 μL of Tris-HCl 50 mM, pH 8 containing 3.5 M urea. The GAGs were extracted by proteolysis with the enzyme Maxatase (4 mg/ml) for 18 hours at 60°C in the presence of Tris-HCl 50 mM, NaCl 0.15 M, pH 8, and non-radioactive carriers 1 mg/ml containing C4S, C6S (Seikagaku Kogyo Co. Tokyo, Japan), DS (Opocrim Laboratories, Modena, Italy) and HS (purified in our laboratory as described by Dietrich & Nader [31]). The GAGs were analyzed by agarose gel electrophoresis as described by Jaques et al. [32] and modified by Dietrich & Dietrich [33]. Agarose slides were exposed to radio-sensitive film for 24 hours and subsequently scanned in a Cyclone device using OptiQuant software (Perkin Elmer). For quantification, the radioactive bands were scraped off the agarose gels, and counted in 5 ml of Ultima Gold (PerkinElmer) in a liquid scintillation spectrometer. Data were expressed as total cpm/μg of protein. The concentration of protein in each sample was determined in an aliquot of the cell fraction using the BCA Protein Assay Kit (Thermo Scientific Inc., IL, USA).

### RNA extraction and real-time reverse transcription-PCR (qPCR)

Total RNA was extracted from ECs after exposure to 4 dyn/cm^2^ SS or 12 dyn/cm^2^ SS for 4 hours, or ECs in static conditions, using TRIzol® Reagent (Ambion Life Technologies). The RNA was spectrophotometrically quantified and used as the template for the reverse transcriptase reaction, using NanoDrop ND-1000 (Thermo Fisher Scientific, EUA). Complementary DNA (cDNA) was reverse transcribed using 2 μg of total RNA and the TaqMan® Reverse Transcription Reagents kit (Applied Biosystems, Foster City, CA, USA). After synthesis, cDNA was transcribed and 500 ng of cDNA was spectrophotometrically measured (260/280 nm) using NanoDrop ND-1000 (Thermo Fisher Scientific, EUA). The quantitative polymerase chain reaction (qPCR) was performed using the reagent Power SYBR® Green PCR Master Mix (Applied Biosystems, USA) and the reactions were performed on a 7500 Real-Time PCR System (Applied Biosystems, USA). For each qPCR reaction, we used 1 μl of template cDNA with specific primers (Invitrogen^TM^, Life Technologies, USA) described in S1 Table in the supplementary information, using the activation cycle: 50°C (2 min), 95°C (10 min), 40 cycles of 95°C (15 sec), 60°C (1 min), and 72°C (30 sec). All reactions were performed in 96-well plates in real time (Applied Biosystems, USA). Data analysis was using the 2^-ΔΔCT^ method [34, 35]. The results are shown using the housekeeping gene GAPDH.

Next the PCR products were column purified using Wizard®RSVGel and PCR cleanup system (Promega, USA) and sequenced using BigDye™ Terminator v3.1 Cycle Sequencing Kit– 4336697 (Applied Biosystems, USA) where each of the amplification primers were used at a concentration of 1 nM to read both DNA strands. The sequencing was performed on an ABI PRISM 3130 automated sequencer (Applied Biosystems, USA), and the sequences were analyzed using the FinchTV® Software v1.4 with reference sequence alignment.

### Statistical analysis

Two-way analysis of variance (ANOVA) or Student's t test were used for comparisons in the shear stress experiments. A P-value less than 0.05 was considered significant. Prism 7 from GraphPad Software (La Jolla, CA) was used for statistical analyses. The data were expressed as means ± standard error of the mean (SEM) for the indicated number of experiments (n).

## Results

### Behavior of endothelial cells after exposure to shear stress

**Fig 1:**
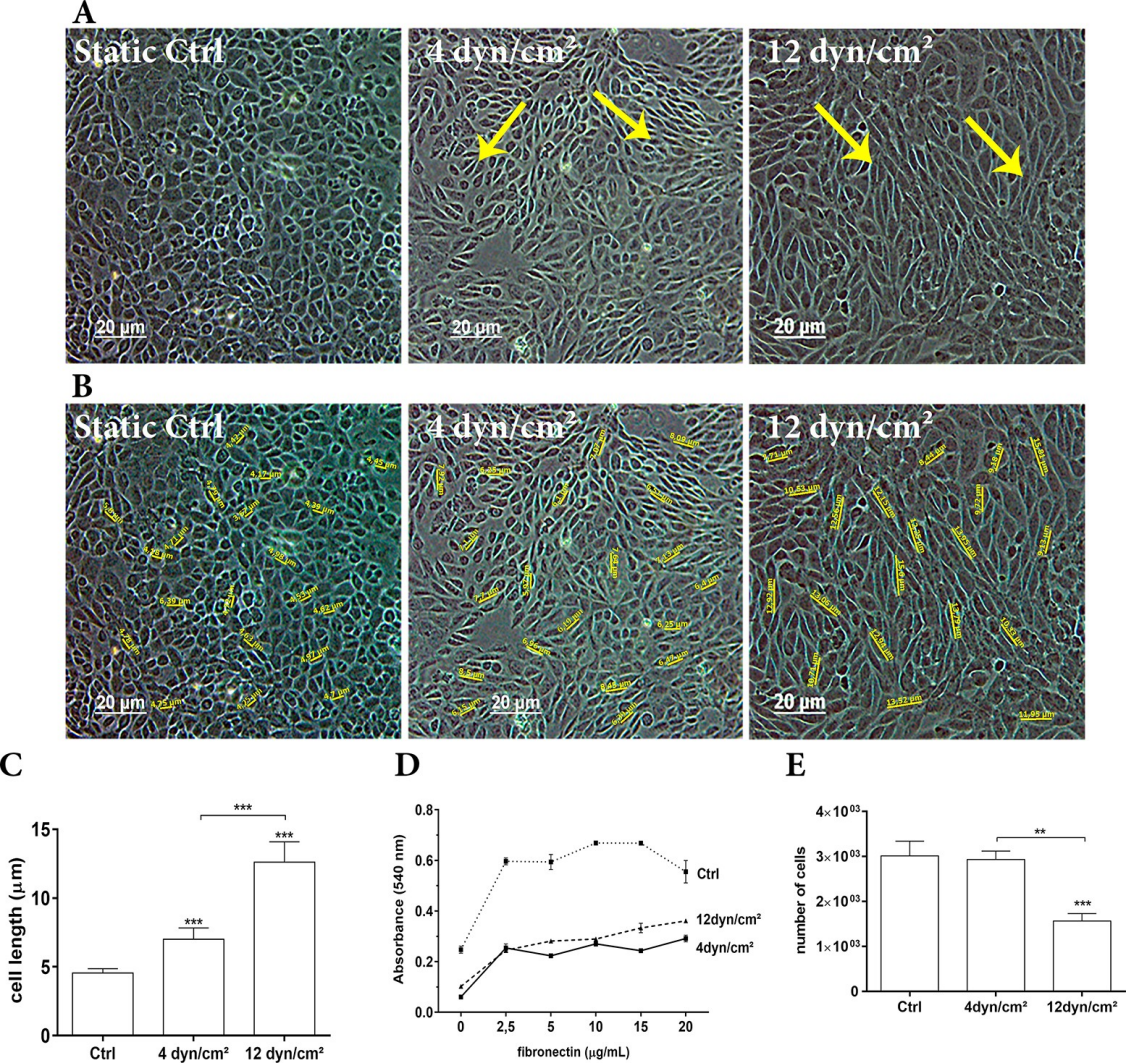
Effects of EC after exposure to shear stress. ECs were exposed to 4 dyn/cm^2^ (pathological flow), 12 dyn/cm^2^ (physiological flow) or were cultured under static conditions. (A) Morphological changes induced by mechanical force leading to re-orientation of cells. (B) The same image with demarcation of endothelial cell length in static culture or when exposed to SS. (C) Graphical representation of endothelial cell length in static culture or when exposed to SS (μm). 40 X magnification. Scale bar = 20 μm. (D) Adhesion assay on fibronectin (0, 2.5, 5, 10, 15 or 20 μg/mL). Values are expressed as absorbance at 540 nm. *p < 0.01; **p < 0.001; ***p < 0.0001 (ANOVA, Bonferroni test). The assays were performed in triplicate. (E) Transwell® migration assay. *p < 0.01; **p < 0.001; ***p < 0.0001 (ANOVA, Bonferroni test). The assays were performed in triplicate.

Bright field microscopy showed that ECs exposed to SS of 4 dyn/cm^2^ or 12 dyn/cm^2^ for 4 h produced morphological features that differed from those typical of static control culture. We observed that, under SS conditions, ECs changed morphology in the direction of the flow course (Fig 1A). Fig 1B displays same morphological features in ECs; however, a demarcation of cell length is demonstrated. Fig 1C shows the graphical representation of ECs length, where we observed that ECs exposed to physiological SS (12 dyn/cm^2^) for 4 h showed an increase of cell length of 2.8-fold when compared to ECs in static conditions and an increase of 1.8-fold when compared to ECs exposed to 4 dyn/cm^2^. ECs exposed to pathological SS (4 dyn/cm^2^) for 4 h showed an increase of 1.5-fold when compared to ECs in static conditions (*P* < 0.0001).

The adhesion assay on fibronectin is presented in Fig 1D, where ECs after exposure to SS presented lower adhesion. EC exposure to 4 dyn/cm^2^ SS showed a decrease of adhesion to fibronectin than those exposed to physiological SS (12 dyn/cm^2^).

EC adhesion was 1.3-fold higher after ECs exposure to physiological SS on 5 μg/mL and 20 μg/mL fibronectin compared to pathological SS, and was 1.3-fold higher after EC exposure to physiological SS on 15 μg/mL fibronectin compared to pathological SS.

These results suggest that ECs exposed to physiological SS of 12 dyn/cm^2^ (atheroprotective) had higher adherence than did those exposed to pathological SS of 4 dyn/cm^2^. Nevertheless, the results also show that ECs maintained in static conditions presented higher adhesion at all concentrations of fibronectin compared to both groups exposed to SS.

To investigate EC migration after exposure to SS, ECs were subjected to a Transwell® migration assay. ECs exposed to physiological SS (12 dyn/cm^2^) for 4 h showed a decrease of 1.9-fold migration (P < 0.0007) than those exposed to 4 dyn/cm^2^ or ECs on static conditions (Fig 1E). Our results demonstrated that physiological SS of 12 dyn/cm^2^ induced a lower migration of ECs compared with ECs exposed to pathological SS of 4 dyn/cm^2^ or ECs in static conditions, suggesting a possible injury of blood vessels and the requirement of cell migration to the local.

#### Angiogenesis assay

**Fig 2:**
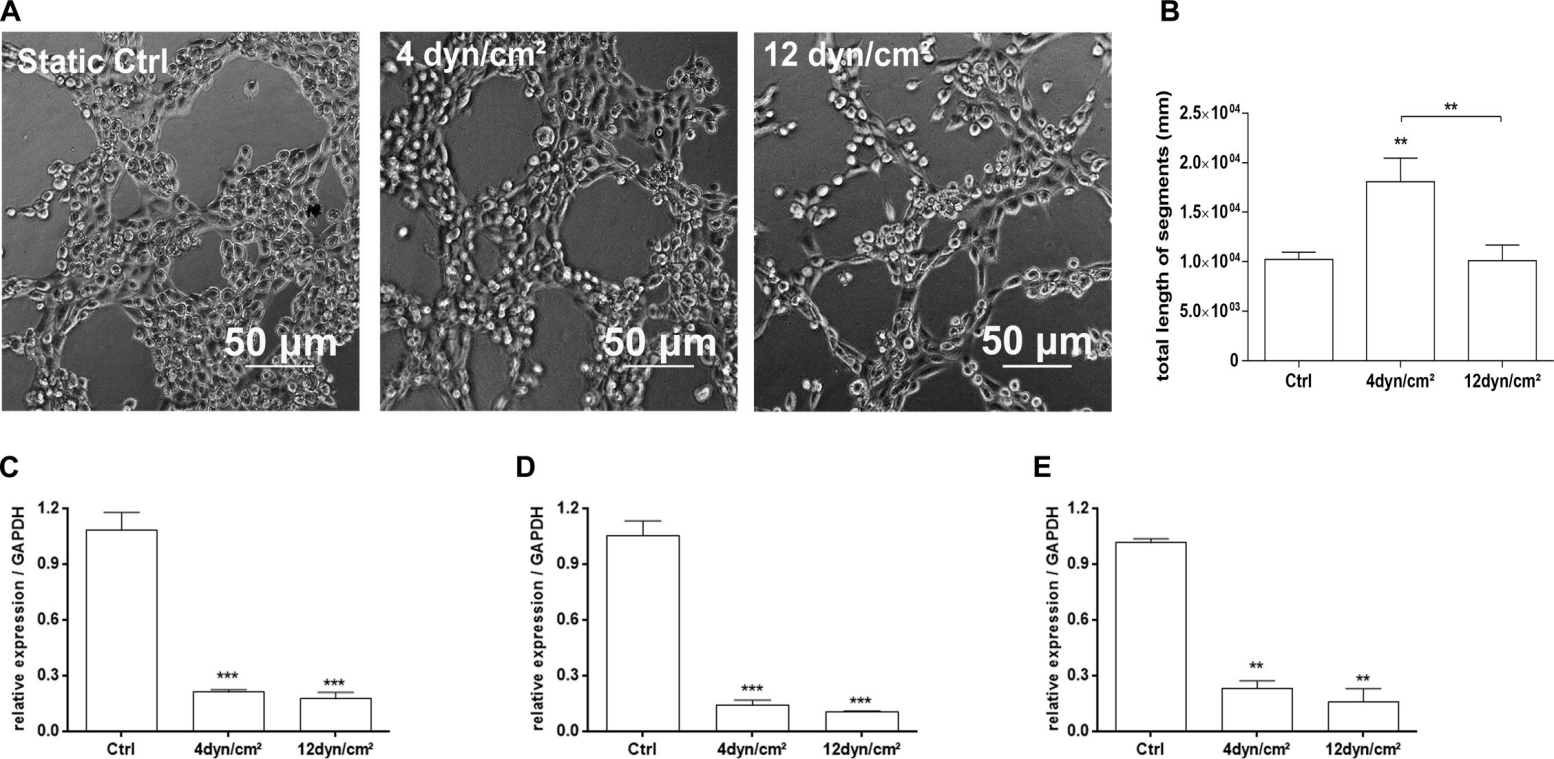
Angiogenesis-related assays of ECs exposed to shear stress. (A) Capillary-like tube formation assay on reconstituted basement membrane was performed on ECs exposed to 4 dyn/cm^2^, 12 dyn/cm^2^ or ECs in static conditions, to assess the ability of these cells to form an organized tubular network. The images were acquired under an inverted light microscope at 40 X magnification. Scale bar = 50 μm. (B) The total length of capillary segments connected forming tubular structures on the Geltrex^TM^ was measure using ImageJ® software as “mm” tube length/cm^2^ area. (C) Analysis of gene expression in qPCR of VEGF-A; (D) Analysis of gene expression in qPCR of TGFβ1 and (E) Analysis of gene expression in qPCR of TGFβ3. *p < 0.01; **p < 0.001; ***p < 0.0001 (ANOVA, Bonferroni test). The assays were performed in triplicate.

For the study of angiogenesis, a capillary tube formation assay was performed using reconstituted basement membrane (Geltrex^TM^, Gibco®—Life Technologies). Fig 2A shows images obtained by inverted light microscopy of the capillary-like tube formation assay using ECs previously subjected to SS. Fig 2B displays the values of the total length (in mm) of segments forming capillary-like tube structures. ECs exposed for 4 h to pathological SS showed 1.8-fold greater capillary-like tube formation (*P* = 0.0044) compared to ECs exposed to physiological SS or ECs in static culture.

The capillary-like tube formation assay corroborates the results of qPCR analysis of angiogenesis-related growth factors, such as VEGF-A (vascular endothelial growth factor A), TGF-β1 (transforming growth factor beta 1) and TGF-β3 (transforming growth factor beta 3). Fig 2C shows the exposition of ECs to SS lead a decrease of VEGF-A gene expression of 5.1-fold in 4 dyn/cm^2^ SS and 6.2-fold in 12 dyn/cm^2^, both compared to ECs in static conditions. Fig 2D shows the exposition of ECs to SS lead a decrease of TGF-β1 gene expression of 7.5-fold in 4 dyn/cm^2^ SS and 10-fold in 12 dyn/cm^2^, both compared to ECs in static conditions. Fig 2E shows the exposition of ECs to SS lead a decrease of TGF-β3 gene expression of 4.4-fold in 4 dyn/cm^2^ SS and 6.3-fold in 12 dyn/cm^2^, both compared to ECs in static conditions.

Taken together, these results demonstrate a slight increase of gene expression of angiogenic growth factors (VEGF-A, TGF-β1 and TGF-β3) in ECs exposed to pathological SS (4 dyn/cm^2^) compared to physiological SS (12 dyn/cm^2^), suggesting a possible increase in angiogenesis, corroborating the results of the capillary-like tube formation assay.

### Evaluation of sulfated glycosaminoglycans present in endothelial cells exposed to shear stress

**Fig 3:**
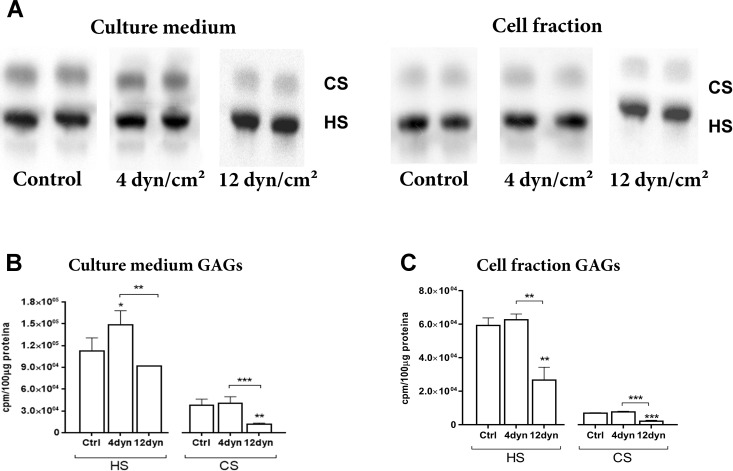
Analysis of sulfated glycosaminoglycans synthesized by ECs exposed to shear stress. After exposure to 4 dyn/cm^2^, 12 dyn/cm^2^ or maintained in static conditions, ECs were metabolically radiolabeled for 18 hours with F12 medium containing [^35^S]-sulfate (150 μCi/ml). After the incubation period, GAGs from the culture medium and cell fraction were extracted, analyzed and quantified. (A) Electrophoretic behavior of the sulfated GAGs present in the culture medium and cell fraction of ECs after exposure to shear stress. (B) Analysis of the absolute values of the HS and CS secreted to the culture medium. (C) Analysis of total GAGs content synthesized by the cells. The results are expressed as scintillation counts per minute normalized by protein quantification of the sample (cpm/100 μg protein). The gels were cut and organized in this image for a better understanding of the results. CS: chondroitin sulfate; HS: heparan sulfate; *p < 0.01; **p < 0.001; ***p < 0.0001 (ANOVA, Bonferroni test). The experiments were performed in duplicate.

After 4 h of exposure to SS of 4 or 12 dyn/cm^2^, or maintained in static conditions, ECs were incubated for 18 h in the presence of 150 μCi/ml [^35^S]-sodium sulfate. After the incubation period, the glycosaminoglycans secreted into the culture medium and glycosaminoglycans present in the cellular fraction (cell and extracellular matrix) were extracted, analyzed and quantified using agarose gel electrophoresis, followed by exposure to radiosensitive film as described in the methods section. All groups analyzed showed a main band that represents heparan sulfate (HS) in both the culture medium and the cellular fraction, as well as an upper band identified as chondroitin sulfate (CS) (Fig 3A).

HS or CS synthesized by ECs and secreted to the culture medium after exposure to 4 dyn/cm^2^ SS, 12 dyn/cm^2^ SS or ECs in static conditions (Fig 3B). After exposure to 4 dyn/cm^2^ SS, ECs showed increased secretion of HS to the culture medium of 1.2-fold compared to ECs exposed to 12 dyn/cm^2^ SS and an increase of 1.6-fold when compared to ECs in static conditions (*P* = 0.0036). The CS secreted to culture medium by the ECs exposed to 12 dyn/cm^2^ SS showed a 3.6-fold decrease compared to those exposed to 4 dyn/cm^2^ SS and a 3.4-fold decrease when compared to ECs in static conditions (*P* = 0.0007).

HS or CS synthesized by ECs and present in the cellular fraction (cell plus extracellular matrix) after exposure to 4 dyn/cm^2^ SS, 12 dyn/cm^2^ SS or ECs in static conditions (Fig 3C). ECs exposed to 12 dyn/cm^2^ SS showed a 2.3-fold decrease compared to those exposed to 4 dyn/cm^2^ SS and a 2.2-fold decrease when compared to ECs in static conditions (*P* = 0.0008). The CS synthesized and present in the cellular fraction of ECs exposed to 12 dyn/cm^2^ SS showed a 3.6-fold decrease compared to those exposed to 4 dyn/cm^2^ SS and a 3.2-fold decrease when compared to ECs in static conditions (*P* < 0.0001).

These results suggest that pathological SS (4 dyn/cm^2^ SS) to ECs leads an increase of the synthesis of HS secreted to culture medium and present in the cell fraction when compared to those exposed to physiological SS (12 dyn/cm^2^). Furthermore, the assay shows that pathological SS causes an increase in the synthesis of CS secreted to culture medium and present in the cell fraction when compared to physiological SS.

### Extracellular matrix and cell surface molecules expression and location in endothelial cells exposed to shear stress

Gene expression measured by qPCR and protein/proteoglycan analyses using immunofluorescence analyzed by confocal microscopy were performed to study the effect of shear stress on cell surface molecules, including syndecan-4 and connexin-43, was well as on ECM molecules such as perlecan.

#### Syndecan-4

**Fig 4:**
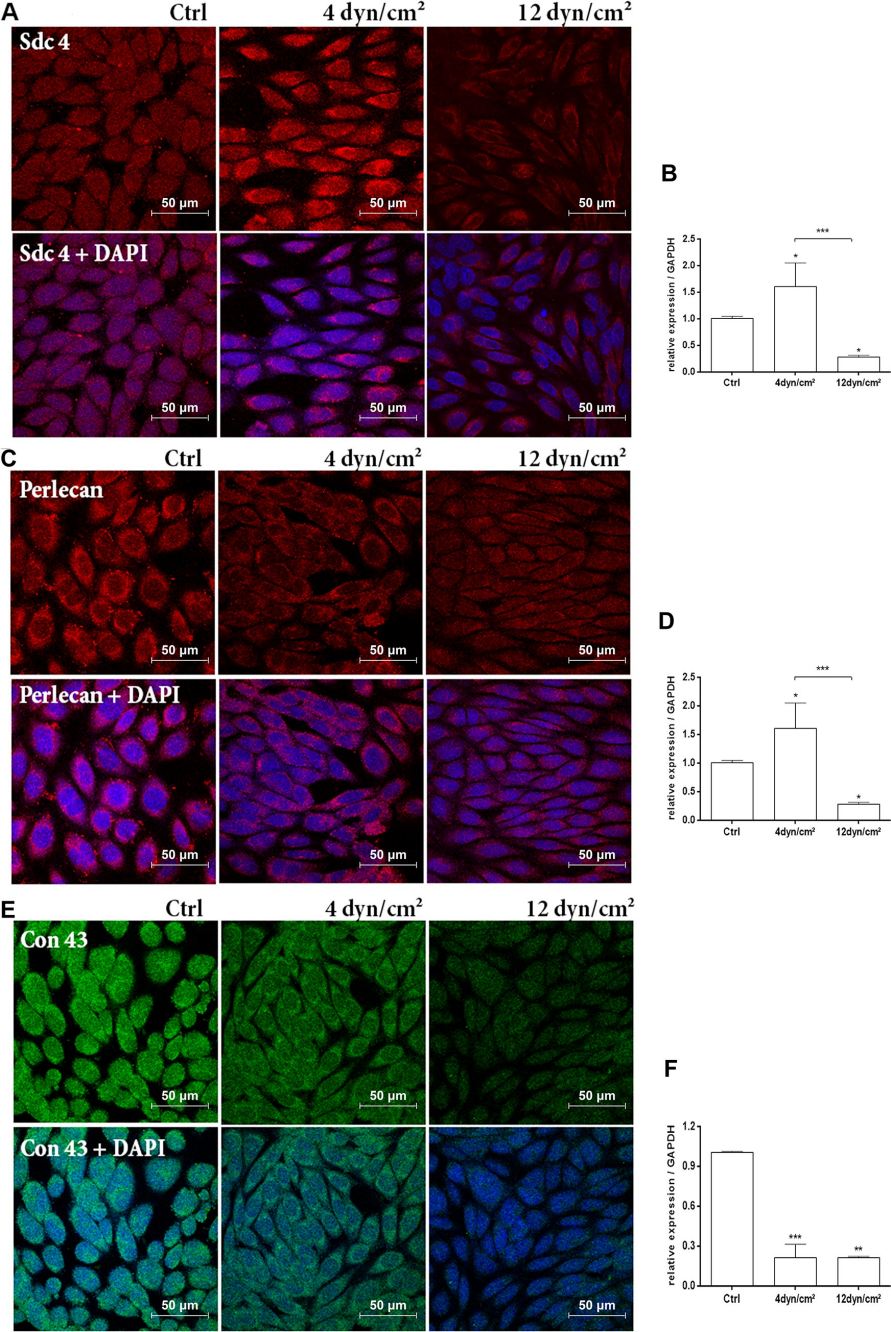
Proteoglycans and connexin immunofluorescence and gene expression of ECs exposed to shear stress (4 dyn/cm^2^ or 12 dyn/cm^2^) or ECs in static conditions. (A) Location of syndecan-4 by immunofluorescence analyzed with confocal microscopy. (B) Analysis of the gene expression of syndecan-4 core protein by qPCR. (C) Location of perlecan by immunofluorescence analyzed with confocal microscopy. (D) Analysis of the gene expression of perlecan core protein by qPCR. (E) Location of Connexin 43 by immunofluorescence using confocal microscopy. (F) Analysis of the gene expression of Connexin 43 by qPCR. Red: Alexa Fluor 594 secondary antibody for staining of syndecan-4 or perlecan; Green: Alexa Fluor 488 secondary antibody for staining of Connexin 43; Blue: DAPI, nuclei staining. 630 X magnification. Scale bar = 50 μm. Sdc4: Syndecan-4. Con43: Connexin 43. RE: Relative expression to GAPDH. *p < 0.01; **p < 0.001; ***p < 0.0001 (ANOVA, Bonferroni test). The assay was performed in triplicate.

Immunofluorescence showed that ECs exposed to pathological SS (4 dyn/cm^2^) tended to show increased staining for Sdc4 at the cell surface when compared with those exposed to physiological SS (12 dyn/cm^2^) (Fig 4A). We also observed that with higher SS, Sdc4 localization was concentrated as clusters at the cell membrane, possibly indicating the presence of these molecules at focal adhesion points.

Real-time PCR of expression of Sdc4 (Fig 4B) showed that ECs exposed to 4 dyn/cm^2^ SS presented an increase of 1.6-fold in Sdc4 expression when compared to ECs in static conditions and an increase of 5.8-fold when compared to 12 dyn/cm^2^ SS (*P* < 0.001). ECs exposed to 12 dyn/cm^2^ SS presented a decrease of 3.7-fold when compared to ECs in static conditions (*P* < 0.001).

#### Perlecan

Perlecan immunofluorescence staining and intensity showed no differences between ECs exposed to 4 and 12 dyn/cm^2^ SS or in static conditions (Fig 4C).

Real-time PCR of expression of perlecan (Fig 4D) showed that ECs exposed to 4 dyn/cm^2^ SS presented an increase 4.4-fold in perlecan expression when compared to ECs in static conditions and an increase of 2.4-fold when compared to 12 dyn/cm^2^ SS (*P* < 0.001). ECs exposed to 12 dyn/cm^2^ presented a decrease of 10.4-fold when compared to ECs in static conditions.

#### Connexin 43 (Con43)

Con43 immunofluorescence showed a decrease of staining in ECs after exposure to SS compared to ECs in static conditions. ECs exposed to pathological SS (4 dyn/cm^2^) presented an increase of staining of Con43 when compared to ECs exposed to physiological SS (12 dyn/cm^2^) (Fig 4E).

Real-time PCR of expression of Con43 (Fig 4F) showed that ECs exposed to 4 dyn/cm^2^ SS or 12 dyn/cm^2^ SS presented a decrease of 4.7-fold in Con43 expression when compared to ECs in static conditions (*P* = 0.0005).

### Gene expression of extracellular matrix molecules by qPCR

**Fig 5:**
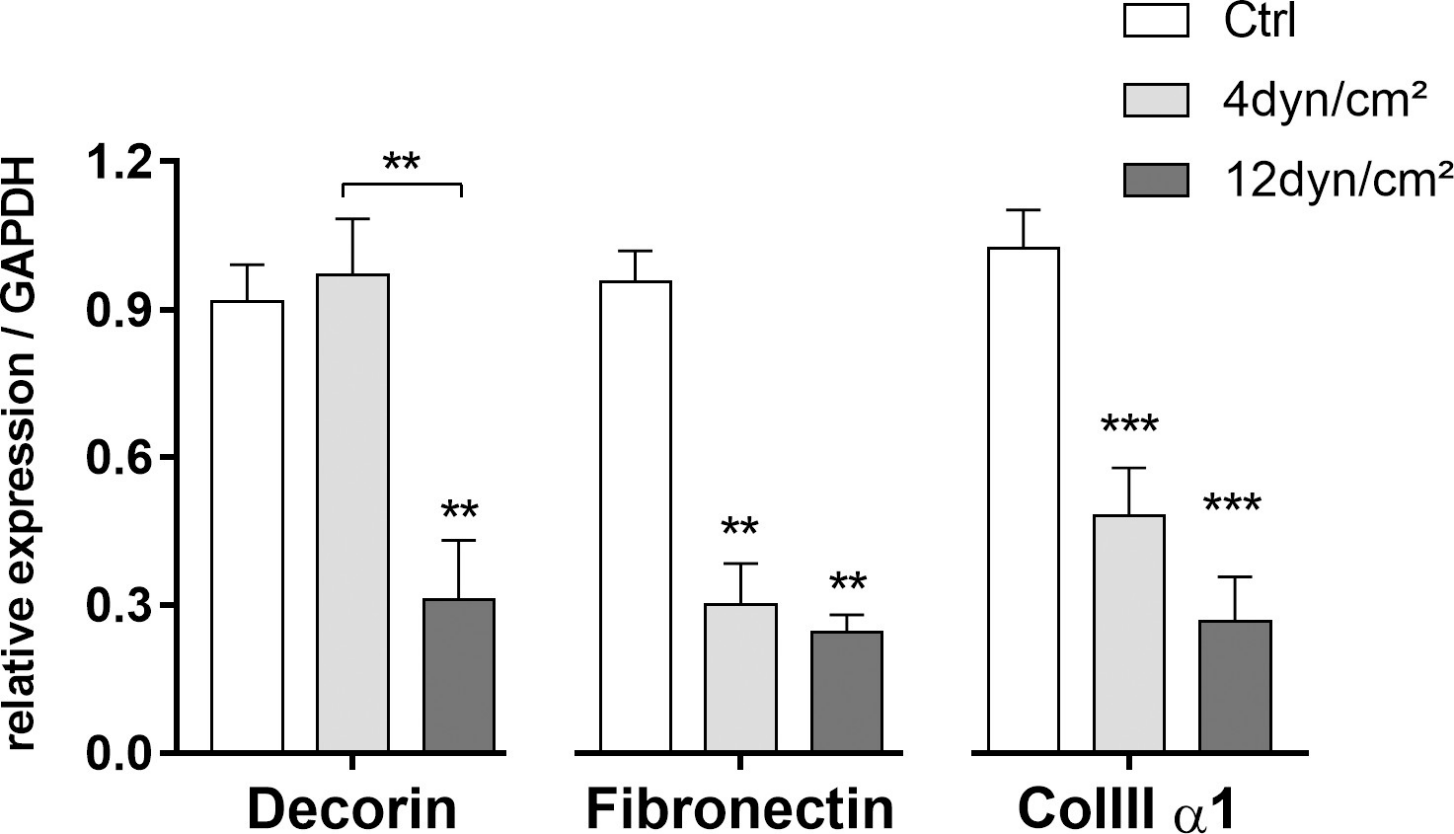
Analysis of gene expression by qPCR from ECs after exposure to shear stress (4 dyn/cm^2^ or 12 dyn/cm^2^) or ECs in static conditions. Analysis of gene expression by qPCR of Decorin core protein, Fibronectin and Collagen IIIα1. RE: Relative expression compared to GAPDH. *p < 0.01; **p < 0.001; ***p < 0.0001 (ANOVA, Bonferroni test). The assay was performed in triplicate.

The analysis of gene expression by qPCR of Decorin, Fibronectin and Col IIIα1 of ECs exposed to SS or in static conditions is presented in Fig 5.

#### Decorin (DCN)

ECs exposed to 4 dyn/cm^2^ SS showed increased decorin expression of 2.4-fold when compared to ECs exposed to 12 dyn/cm^2^ SS (*P* = 0.0011). ECs exposed to 12 dyn/cm^2^ showed a decrease decorin expression of 2.9-fold of gene expression of ECs in static conditions (*P* < 0.0001). Therefore, ECs exposed to pathological SS showed a higher expression of decorin when compared to ECs exposed to physiological SS or static conditions.

#### Fibronectin

ECs exposed to 4 dyn/cm^2^ SS showed a decrease of fibronectin expression of 3.2-fold when compared to ECs in static conditions (*P* = 0.0028). ECs exposed to 12 dyn/cm^2^ showed a decrease fibronectin expression of 3.9-fold of gene expression of ECs in static conditions (*P* = 0.0028). Therefore, ECs exposed to pathological SS showed higher expression of fibronectin when compared to ECs exposed to physiological SS.

#### Col III α1

ECs exposed to 4 dyn/cm^2^ SS showed a decrease of Col III α1 expression of 2.1-fold when compared to ECs in static conditions (*P* < 0.0001). ECs exposed to 12 dyn/cm^2^ showed a decrease Col III α1 expression of 3.8-fold of gene expression of ECs in static conditions (*P* < 0.0001). Therefore, ECs exposed to pathological SS showed higher expression of Col III α1 when compared to ECs exposed to physiological SS.

## Discussion

When ECs are subjected to mechanical forces, a set of responses is rapidly generated, including proliferation, migration, morphology and adherence. When ECs were subjected to SS in vitro, the cells became directed towards the flow and become more elongated. The SS exerted in the flow direction oriented cell surface molecules and reoriented their migration route in the direction of the SS [3, 36]. This mechanical force occur in the vasculature and is probably the most important phenomena implicated in alterations of the adhesion structures present in ECs, triggering intracellular signaling cascades [37]. In this respect, one of the most abundant proteins in the ECM is fibronectin, an adhesion glycoprotein involved in the maintenance of blood vessel morphology. When immobilized on any surface, fibronectin promotes adhesion and cell accommodation [38]. Our results demonstrated that ECs exposed to physiological SS showed higher cell adhesion than those exposed to pathological SS. Therefore, physiological SS promotes cellular responses implicated in cell-matrix adhesion, leading to an increase of anchoring [39] and a subsequent decrease of cell migration.

Angiogenesis has a central role in the metabolism not only of normal tissue (during embryonic and fetal development, or wound healing processes), but also of tissues under pathological conditions, as in diabetic retinopathy, age-related macular degeneration, retinopathy of prematurity, or tumors [40, 41]. It is relevant that hemodynamic forces are directly related to the regulation of angiogenesis [40, 42]. Studies have demonstrated direct modulatory mechanical force effects of SS and cell stretch on adhesion, migration and morphogenesis of ECs [16, 41, 43]. Lesions in the vascular endothelium are common several processes such as inflammation and atherosclerosis where SS usually is low and/or is turbulent. After injury, the endothelium starts a healing process that is closely associated with the migration of ECs, where mechanical forces change the migration speed of ECs, causing the injured site to heal faster [44, 45]. The pathological environment promotes migration and lower adhesion, as previously shown [46]. These results demonstrate that the wound healing process is modulated by mechanical forces to which ECs are exposed, promoting faster closure of the lesion. In this study, we observed a positive modulatory effect on capillary tube formation of ECs exposed to 12 dyn/cm^2^ SS when compared to those exposed to 4 dyn/cm^2^ SS. These results demonstrate that exposure of ECs to physiological SS leads to increased capillary-like tube formation, supporting the notion that SS has a direct modulatory effect on adhesion.

Altered mechanical forces may induce cardiovascular diseases such as hypertension, thrombosis and atherosclerosis. The endothelial glycocalyx acts as a mechanosensor, primarily composed of HS, CS and hyaluronic acid that interacts with cytoskeleton of ECs and is responsible for mechanical deformation [5]. The activity of mechanical forces on GAGs expression influences endothelial behavior. The glycocalyx, present primarily as HS at the cell surface in regions where the endothelium is exposed to SS, mediates atheroprotection; however, in areas with low or turbulent SS, the HS content is altered, and the site becomes susceptible to atherosclerosis [36, 47]. The expression of glycocalyx components are regulated by hemodynamic forces [47].

Proteoglycans from glycocalyx and extracellular matrix, including heparan sulfate proteoglycans (HSPG), are widely present and distributed in healthy endothelium; however, when the endothelium is compromised, there is a greater need of repair and thus greater expression of these molecules. Our results demonstrate precisely this: that pathological SS induces increases in the amounts of HS and CS in ECs compared to those exposed to physiological SS. Chondroitin sulfate is present in ECM, cell surfaces and ECs granules, and is found in increased proportions in vascular diseases. The transmembrane CS proteoglycans secreted by ECs (CD44 and thrombomodulin) may release their proteoglycan ectodomains to create an anti-coagulant surface that is released to the circulation. The secretory granule proteoglycan serglycin contains CS and heparin and is important for the function of these vesicles; once secreted, it enhances anticoagulant activity [48]. Extracellular CS proteoglycans such as versican and biglycan are pro-atherogenic PGs because they have a holding capacity for lipoprotein and growth factors [49]. In this study, ECs exposed to pathological shear stress synthesized higher amounts of CS when compared to ECs exposed to physiological shear stress.

Sdc4, a cell surface HSPG, plays a role in tissue repair such that its expression is increased at the time of injury to induce anti-thrombotic and anti-atherogenic actions. The literature suggests that high peaks of Sdc4 are found in plasma of patients with acute myocardial infarction, and there is increased expression in repaired areas of injured cardiac tissue, suggesting that Sdc4 is overexpressed under pathological conditions [50]. Therefore, Sdc4 expression is increased, playing anti-thrombotic and anti-atherogenic roles. Both results regarding HS synthesis and Sdc4 expression are in accordance with results the literature, because we demonstrated that ECs exposed to pathological SS showed increased expression of Sdc4 compared to ECs exposed to physiological SS.

Perlecan is a major HSPG in the ECM, more specifically in the basement membrane. It is important for the development of atherosclerosis because it has areas where the core protein can capture lipoproteins or cell adhesion molecules [51]. In addition, perlecan can bind through its HS chains, capturing growth factors such as FDF-2, PDGF and VEGF, thereby stimulating angiogenesis. Perlecan also has a C-terminal portion, a potent angiostatic agent called endorepelin that is released by a proteolytic process [52]. Angiogenic stimuli and hemodynamic forces often increase perlecan deposition at the basal lamina, stimulating angiogenesis and cell proliferation. We demonstrated that ECs exposed to pathological SS showed higher expression of perlecan when compared to those exposed to physiological SS. ECs under pathological conditions showed higher amounts of perlecan, probably as an attempt restore the normal state where ECs present anti-thrombotic and angiostatic profiles.

In a healthy endothelium, ECM is composed of a large quantity of laminin, type IV collagen and proteoglycans, and small amounts of fibronectin. By contrast, in damaged tissue forming regions of atherosclerotic plaque or after an injury, accumulation of fibronectin can be observed [53]. In the present study, fibronectin showed a decrease in both group, physiological and pathological, however, there is a slight increase of expression in ECs exposed to pathological SS when compared to those exposed to physiological SS. These results suggest that fibronectin increases its expression where there is a need for tissue repair, wound healing and remodeling of ECM. Decorin is present in the ECM with intrinsic anti-tumorigenic and anti-angiogenic properties. Decorin also protects the microenvironment from inflammation [54]. In the present study, we observed higher expression of decorin in ECs exposed to pathological SS when compared to those exposed to physiological SS, suggesting an inflammatory environment.

During tissue repair, an increase in type I and type III collagen fibers can be observed, both having a central role in the repair process. In the present study, higher gene expression of collagen type IIIα1 was found a decrease in both group, physiological and pathological, however, there is a slight increase of expression in ECs exposed to pathological SS compared to those exposed to physiological SS.

Other molecules involved on cell-cell attachment that have a role in mechanotransduction are the connexins. They are expressed in healthy endothelium, primarily connexin 37 (Con37) and connexin 40 (Con40); however, in atherosclerotic regions, these proteins are barely detectable. Nevertheless, connexin 43 (Con43) can be detected in regions susceptible to the formation of atherosclerotic plaques [55]. The results of the present study showed a tendency to an increased gene expression of Con43 when ECs were exposed to the pathological shear stress compared to the physiological shear stress. The immunofluorescence showed an increased labeling of the Con43 at ECs when these cells were exposed to the pathological shear stress compared to the physiological shear stress.

## Conclusions

Shear stress influences endothelial cell triggering of intracellular signals. This phenomenon is known as mechanotransduction, and it can impact remodeling of the extracellular matrix, cell behavior and gene expression of various molecules related to vascular biology.

Several mechanisms of response were observed in endothelial cells exposed to pathological shear stress compared to those exposed to physiological shear stress, implying alterations of homeostasis. The changes in hemodynamic forces affect the microenvironment and these pathological stimuli may cause inflammation, angiogenesis, apoptosis, modifications of cellular adhesion and migration, and extracellular matrix remodeling at the site. All these effects predispose vessels to the development of atherosclerotic lesions, stiffening of arterial walls and other vessel disorders. Consequently, understanding of the impact of altered shear stress on endothelial cells is essential for better comprehension of cardiovascular diseases in order to provide new parameters and interpretations for the understanding of cardiovascular diseases, including atherosclerosis, hypertension, aneurysm and thrombosis.

## Supporting information

S1 Fig Images of negative control of immunofluorescence assay. (TIF)

S1 Table This Oligonucleotides sequences used in real time PCR. (TIF)

S1 Raw images (TIF)
