# Supplementary figures and images for: Altered shear stress on endothelial cells leads to remodeling of extracellular matrix and induction of angiogenesis
Source: PLoS One. 2020 Nov 19;15(11):e0241040. doi: 10.1371/journal.pone.0241040 (PMC7676693; doi:10.1371/journal.pone.0241040)

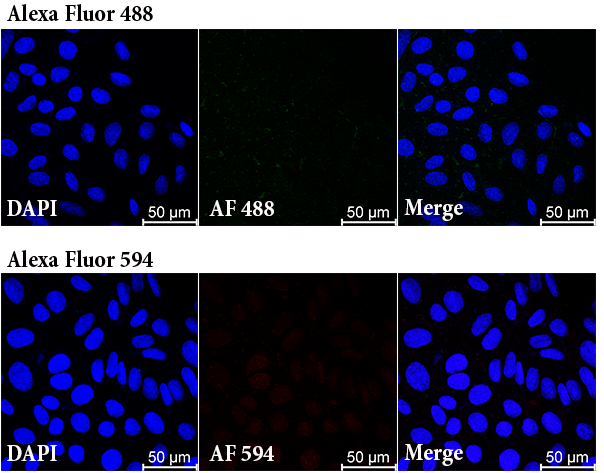

Supplement: S1 Fig — (TIF) [file pone.0241040.s001.tif]

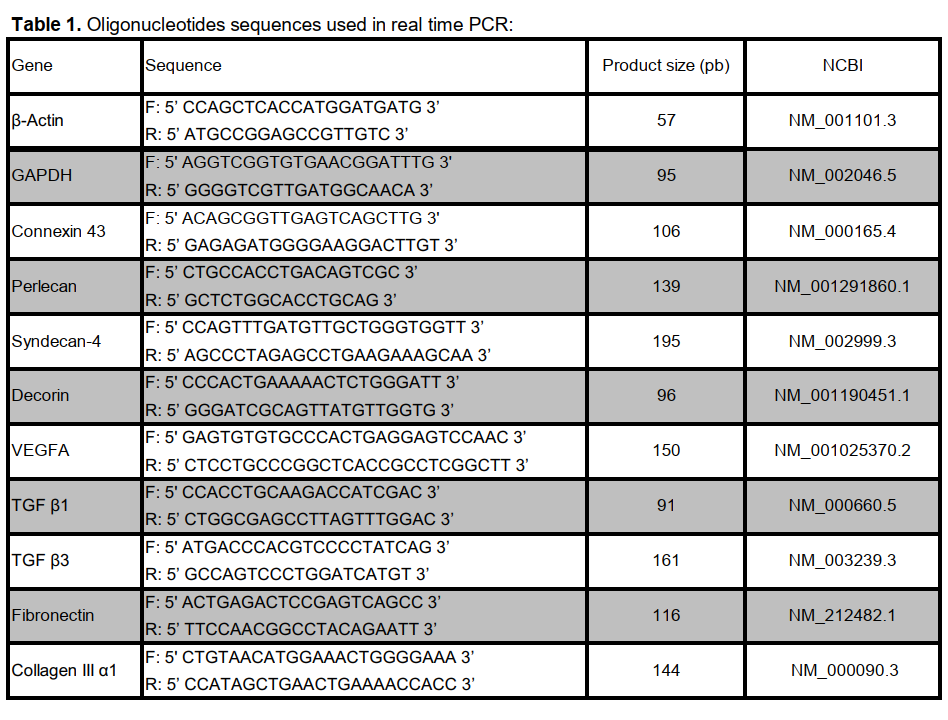

Supplement: S1 Table — (TIF) [file pone.0241040.s002.tif]

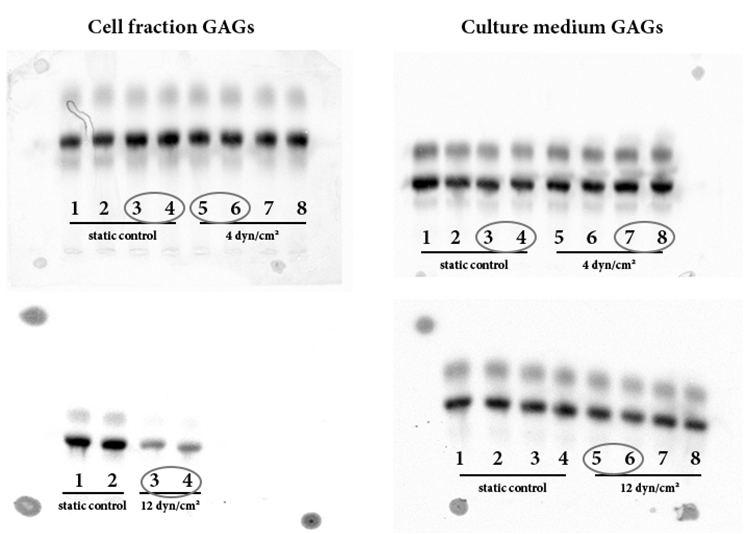

Supplement: S1 Raw images — (TIF) [file pone.0241040.s003.tif]
